# A Novel Fuzzy PID Congestion Control Model Based on Cuckoo Search in WSNs

**DOI:** 10.3390/s20071862

**Published:** 2020-03-27

**Authors:** Lin Lin, You Shi, Jinfu Chen, Sher Ali

**Affiliations:** 1 School of Computer Science and Communication Engineering, Jiangsu University, Zhenjiang 212013, China; linlin@ujs.edu.cn (L.L.); jinfuchen@ujs.edu.cn (J.C.); malaksherkhan@yahoo.com (S.A.); 2 Jiangsu Key Laboratory of Security Technology for Industrial Cyberspace, Jiangsu University, Zhenjiang 212013, China

**Keywords:** proportional integral derivative, cuckoo search, congestion control, wireless sensor networks

## Abstract

Wireless Sensor Networks (WSNs) consist of multiple sensor nodes, each of which has the ability to collect, receive and send data. However, irregular data sources can lead to severe network congestion. To solve this problem, the Proportional Integral Derivative (PID) controller is introduced into the congestion control mechanism to control the queue length of messages in nodes. By running the PID algorithm on cluster head nodes, the effective collection of sensor data is realized. In addition, a fuzzy control algorithm is proposed to solve the problems of slow parameter optimization, limited adaptive ability and poor optimization precision of traditional PID controller. However, the parameter selection of the fuzzy control algorithm relies too much on expert experience and has certain limitations. Therefore, this manuscript proposes the Cuckoo Fuzzy-PID Controller (CFPID), whose core idea is to apply the cuckoo search algorithm to optimize the fuzzy PID controller’s quantization factor and PID parameter increment. Simulation results show that in comparison with the existing methods, the instantaneous queue length and real-time packet loss rate of CFPID are better.

## 1. Introduction

Wireless Sensor Networks (WSNs) are one of the important technologies in recent times due to their widespread applications e.g., military, smart phones, disaster management, health care monitoring and other surveillance systems. In these widespread applications, WSNs may face many challenges, in which network congestion is the prominent one [1]. WSNs can connect network nodes in series to transmit data in a “carry-send” mode where each node has the ability to collect, receive and send data, autonomously. WSNs can potentially improve the transmission date volume and throughput of the network [2]. In addition, the deployment area and the number of nodes is generally very large in WSNs. Furthermore, there is no time limit for data acquisition and transmission, so possibly the network nodes may receive a large amount of data in an instant. If such a high quantity of data arrive at each receiving node instantaneously, a huge burden will be caused on the receiving nodes [3]. When the data receiving rate is not synchronized with the data sending rate, the message queue at the node will be filled rapidly, leading to network congestion.

Serious network congestion will greatly affect the data transmission in WSNs. When network congestion occurs, the data at the node cannot be sent out in time, and other data cannot enter the message queue in the node. The message will be continuously discarded and delayed, which will result in increased data loss, increased transmission delay, reduced network throughput and lowered quality of service of the network. In addition, being in state of high load and full queue for long time can significantly reduce the service life of a node [4]. Therefore, an effective solution for the problem of network congestion is very important.

**Figure 1:**
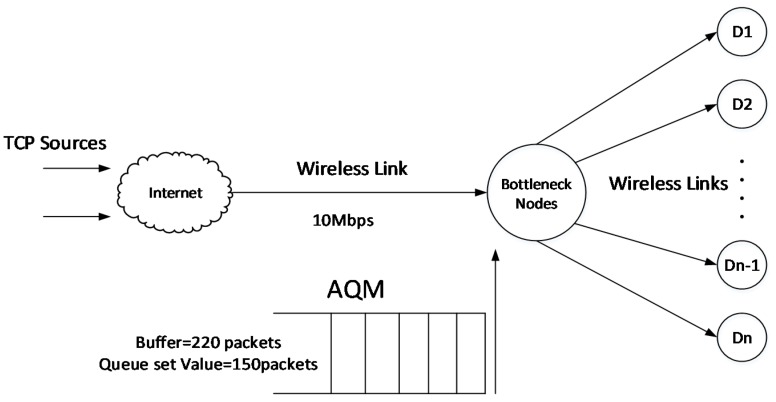
TCP Network Model.

For the network model, we model the Transmission Control Protocol (TCP) network using the network topology shown in the Figure 1. The TCP source sends the data from the Internet to each bottleneck node through the wireless router. When an acknowledgement packet is received, each source increases its transmission rate, eventually causing the bottleneck node’s capacity to be exceeded and congestion is inevitable. According to the network resources of the node itself, the message queue length inside the bottleneck node is adjusted by using the active queue management mechanism.

Cho [5] presented an efficient neural network Active Queue Management (AQM) system as a queue controller. The recurrent neural network has a Multi-layer Perceptron-Infinite Impulse Response (MLP-IIR) structure. Three distinct neural AQMs are trained under different network scenarios involving traffic levels. Selecting one of three neural AQMs is based on posterior probability history of traffic level. Liu [6] introduced a new type of neural networks controller based on PSD (proportion, sum and differential) to improve the lack of fixed gain in single neuron adaptive PID. Li [7] presents extensive comparative simulation results for four neural AQM schemes, namely, Neuron PID, Adaptive Neuron AQM (AN-AQM), Fuzzy Assisted PID controller based on Neuron Network (FAPIDNN), Neuron Reinforcement Learning (NRL), versus three traditional AQM schemes together with a modified PI scheme named Intelligent Adaptive Proportional Integral (IAPI) over a wide range of conditions and scenarios.

## 2. Related Works

Sensor networks with dense distribution of nodes, are usually divided into clusters according to regions. Each cluster has a cluster head, which is used for data collection and forwarding within the cluster. Cluster heads communicate with each other and can be rotated. The designed algorithm is usually placed on the cluster head nodes to solve the problem of data congestion. For the congestion problem of WSNs, researchers have proposed many congestion control mechanisms. Demura [8] proposed a protocol to send data over less crowded paths. Li [9] proposed time-data-driven sleep scheduling and spatial-data-driven anomaly detection approaches to reduce data redundancy. Rajeswari [10] proposed an improved traffic generation mechanism based on TCP/IP protocol to alleviate network congestion. To reduce energy consumption and data loss due to network congestion, Sangeetha [11] proposed an energy-saving congestion control method which periodically adjusts the degree and topology of sensor nodes according to a certain time interval. Paranjape [12] proposed a congestion control technique for intra-cluster congestion control where cluster heads actively monitor the congestion according to the information of traffic intensity, buffer occupancy and number of competitors.

The data collection in WSNs is not bounded by time limit and data quantity, that is, the data collection is irregular, and the network topology changes constantly when the nodes move. In such kind of dynamic conditions, the congestion situation of network node is more complicated. Therefore, an efficient and adaptive congestion control scheme is needed. To this end, Zhang [13] proposed the Proportional Integral Derivative (PID) controller to the network congestion control problem. The PID controller has the advantages of strong adaptability, simple operation and high efficiency, which can effectively reduce the length of message queue and limit network congestion. However, the PID controller itself is not perfect, as it has many defects. The predecessors have proposed many kinds of PID controller improvement schemes, but still have the insufficiencies when it is applied to the WSNs. Yan [14] combined the credit allocation Cerebellum Model Articulation Controller (CACMAC) with the PID controller to further improve the measurement effect, but did not consider that the parameters of the PID controller cannot be changed according to the change of the input quantity, which reduces the credibility of the later data. Mahdi [15] presented an improved PID controller, which improves the control effect and reduces the energy consumption without affecting the transmission link quality. However, this method neglects the problem of low precision in PID controller under the control environment with high requirements. Using the excellent global search ability of the meta-heuristic algorithm to improve the adaptive ability of PID controller. Agarwal [16] combined the meta-heuristic Grey Wolf optimization algorithm with PID controller. However, it also ignores the problem that it will fall into the local optimal accuracy problem in later stage. Pradhan [17] put forward the nonlinear Autoregressive Moving Average (ARMA) algorithm to PID control to realize its parameter self-tuning, but the convergence speed of the ARMA itself needs to be improved. Morawski [18] proposed a data transfer method based on evolutionary game among network nodes. In this method, the nodes do not form a fixed path. The set of nodes participating in the data transfer automatically adjusts to the current network condition by playing a game to obtain a low-cost routing option. However, this method of controlling the direction of node movement may not be suitable for highly random scenarios. Mast [19] presents a method to optimize the PID controller by concave-convex programming and to optimize the design problem by means of gridding. However, the complexity of gridding will increase with the increase of control parameters. Ioannou [20] described in detail the node localization protocol in wireless networks in document. Therefore, the application of PID Controller in congestion control of WSNs needs to consider the problems of low adaptive ability and low calculation accuracy.

Aiming at the defects of PID Controller, we propose the Cuckoo Fuzzy-PID Controller (CFPID), whose core idea is to apply the Cuckoo search algorithm to optimize the fuzzy PID controller’s quantization factor and PID parameter increment. It can effectively adjust and control the congestion in WSNs by its fast convergence ability. In addition, the parameters of CFPID controller can be adjusted by means of the Fuzzy Control Algorithm to improve its adaptive ability. Then, according to the real-time packet loss rate calculated by the mode and PID control, the on-line optimization is carried out by using the Cuckoo search to obtain the optimal precision of the loss probability, thus controlling the instantaneous queue length. This mechanism improves the adaptive ability and accuracy of the control system.

## 3. Fuzzy PID Congestion Control Model Based on Cuckoo Search

We introduce the traditional PID controller into the congestion control of WSNs, and calculate the instantaneous queue length and real-time packet loss rate of message queue. Because of the irregular network environment and data collection, the controller has strong self-adaptive ability. We combine the fuzzy control with the traditional PID controller to realize its parameters self-tuning. The real-time packet loss rate is recalculated, and based on this; a meta-heuristic algorithm with excellent global search ability and fast convergence speed is introduced to optimize the calculation accuracy of the real-time packet loss rate. The proposed algorithm is then used to calculate the real-time packet loss rate of the Cuckoo search. Finally, the length of the instantaneous queue is controlled according to the packet loss rate to achieve the effect of congestion control.

In light of the above ideas, we propose the Cuckoo Fuzzy-PID Controller (CFPID) model, which is divided into two steps: the design of the Fuzzy-PID Controller (FPID) with the queue length of the nodes as the control object, and the optimization of the FPID by the Cuckoo search.

### 3.1. Traditional PID Network Congestion Controller in WSNs

PID is a classical control algorithm which transforms all kinds of algorithms of the active queue management system into the controller. The goal of this algorithm is to keep the value of the control object near the expected value [21]. Compared with the traditional active queue management method, the PID controller has strong robustness and simple model structure which can make the control effect more stable. The principle of PID controller is to combine the proportion of deviation (P), integral (I) and differential (D) to control the controlled object effectively, which makes the controller design more flexible and more selective [22].

**Figure 2:**
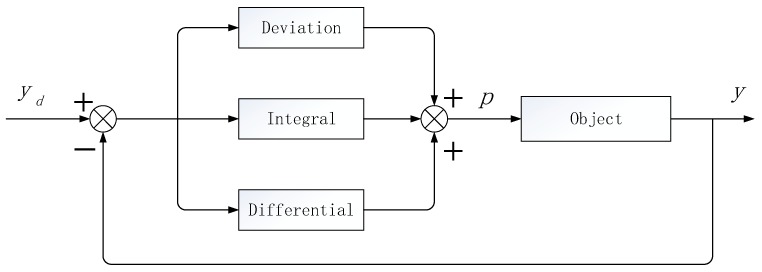
Proportional Integral Derivative (PID) control system schematic diagram.

We apply the PID controller to WSNs, and take the queue length as the control object. After the adjustment of proportion, integral and differential, the message dropping probability is obtained, and the queue length is controlled by the probability of packet dropping to control the degree of congestion. The PID control schematic diagram is shown in Figure 2.

In Figure 1, $y_{d}$ is the expected queue length, y is the instantaneous queue length, p is the message drop probability, and e is the control deviation variable of the queue length which can be expressed as in Equation (1).

(1) $$e=y_{d}-y$$

According to Figure 1, the expected queue length $y_{d}$ is the input and the packet loss rate p is the output to control the instantaneous queue length.

In the process of using PID controller to control the queue length, the function of each control link is as follows.

(1) Proportional control: Once the deviation signal *e* is generated; the control system will immediately reflect it in proportion, the fastest speed to reduce the deviation.
(2) Integral control: It is mainly used to eliminate the steady-state error and reduce the error rate of the control system. The effect of integral control depends on the integral time constant t in such a way that the smaller the t, the more obvious will be the effect of integral control.
(3) Differential control: Differential control can reflect the variation rate and trend of deviation signal. A correction signal is introduced to speed up the operation of the system, and reduce the adjustment time before the deviation signal becomes too large.

Therefore, the control law of the PID controller can be expressed as Equation (2).

(2) $$p\left(k\right)=k_{p}e\left(k\right)+k_{i}T\overset{k}{\underset{j=0}{\sum }}e\left(i\right)+k_{d}\left[\frac{e\left(k\right)-e\left(k-1\right)}{T}\right]$$

The incremental form of p(k) can be expressed as Equation (3).

(3) $$\Delta p\left(k\right)=k_{p}\left[e\left(k\right)\left(1+\frac{T}{T_{i}}+\frac{T_{d}}{T}\right)-e\left(k-1\right)\left(1+\frac{2T_{d}}{T}\right)+e\left(k-2\right)\frac{T_{d}}{T}\right]$$

where, $k_{p}$ is proportional coefficient, $k_{i}$ is integral coefficient, $k_{d}$ is differential coefficient, T is sampling time, $T_{i}=\frac{k_{p}}{k_{i}}$ and $T_{d}=\frac{k_{d}}{k_{p}}$.

### 3.2. Design of Congestion Controller (FPID) Based on Modulus and PID

In the traditional PID controller design, the proportional, integral and differential parameters are fixed while the adaptive adjustment ability is lacking [23]. In view of this flaw, this article uses the mold and the control technology to add the adjustment to the PID controller’s parameter.

**Figure 3:**
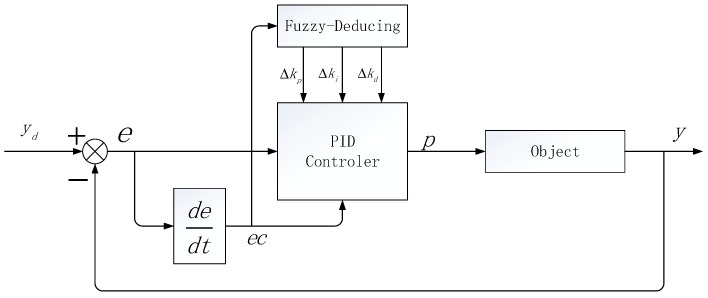
Fuzzy Proportional Integral Derivative (FPID) controller schematic diagram.

The fuzzy control does not need the precise mathematical model of the controlled object, and can be well optimized for the complex control situation of nonlinear change and real-time change [24]. The main work of fuzzy control is to use fuzzy rules to fuzzy control objects and fuzzy reasoning. In this manuscript, we first introduce the mold and control into the PID controller to optimize its parameters, and get the increment of the PID parameters through the mode and rule and the mode and reasoning. Then, new PID parameters are obtained by weighting the initial parameters, so as to realize the parameter self-tuning optimization function of PID. The module and PID control schematic diagram are shown in Figure 3.

Where *e* is the deviation of the queue length in the network node, *ec* is the deviation rate, and $\Delta k_{p}$, $\Delta k_{i}$, $\Delta k_{d}$ are the parameter increments obtained by means of a fuzzy inference. The *e* and *ec* are used as the inputs of the die and the controller while $\Delta k_{p}$, $\Delta k_{i}$ and $\Delta k_{d}$ are used as outputs which result in the final FPID tuning parameter and are expressed as Equations (4)–(6).

(4) $$k_{p}=k_{p0}+\Delta k_{p}$$

(5) $$k_{i}=k_{i0}+\Delta k_{i}$$

(6) $$k_{d}=k_{d0}+\Delta k_{d}$$

where, $k_{p0}$, $k_{i0}$ and $k_{d0}$ are the initial values of the proportional–integral–differential parameters. According to the characteristics of the control object and the performance requirements of the control system, the tuning parameters of the PID controller cannot depend on the mathematical model of the controlled object. According to the regulation law of each link, repeated test, and set $k_{p0}=0.00129$, $k_{i0}=0.00222$, $k_{d0}=0.00095$. The parameter increments $\Delta k_{p}$, $\Delta k_{i}$ and $\Delta k_{d}$ are deduced by the fuzzy rule and model.

#### 3.2.1. The Model and Rule of FPID Controller

In the process of parameter setting, we need to consider the relationship between the parameters. Therefore, the requirements for $\Delta k_{p}$, $\Delta k_{i}$ and $\Delta k_{d}$ are as follows:

(1) When the absolute value of the message queue length offset in the network node |*e*| is large, $\Delta k_{p}$ is a positive number, that is, increase in $k_{p}$ will improve the response speed of regulation; To prevent the occurrence of large overshoot, usually set $\Delta k_{i}=0$. To prevent the differential saturation caused by the instantaneous change of the queue length deviation *e*, $\Delta k_{d}$ should be smaller than the control range.
(2) If |*e*| and |*ec*| are of medium size, then $\Delta k_{i}$ and $\Delta k_{d}$ are of moderate value, which makes the system having small overshoot.
(3) If |*e*| is small, then $\Delta k_{p}$ and $\Delta k_{i}$ take positive numbers, that is, increase in the values of $k_{p}$ and $k_{i}$ will result in good stability of the system.

We set the ambiguity of *e*, *ec*, $\Delta k_{p}$, $\Delta k_{i}$ and $\Delta k_{d}$ as (−3, 3), (−3, 3), (−3, 3), (−0.3, 0.3) and (−0.3, 0.3), respectively. Furthermore, the input and the output variables are divided into seven grades: NB (large negative number), NM (negative number), NS (small negative number), Z (zero), PS (small positive number), PM (positive number), PB (large positive number). In addition, corresponding quantization factors are taken as $k_{e}=0.5$ and $k_{ec}=1$ while the corresponding scaling factors are taken as $k_{\Delta p}=1,\;k_{\Delta i}=1$ and $k_{\Delta d}=1$.

In order to improve the control effect, we combine the triangular membership function with sigmoid membership function.

The analytic function of the triangular membership function can be represented as Equation (7).

(7) $$\mu \left(x\right)\{\begin{array}{c} \frac{x-a}{b-x},\;a\leq x\leq b\\ \frac{c-x}{c-b},\;b\leq x\leq c \end{array}$$

While the analytic function of the sigmoid membership function takes the following form:

(8) $$\mu \left(x\right)=\frac{1}{1+e^{-a\left(x-c\right)}}$$

**Figure 4:**
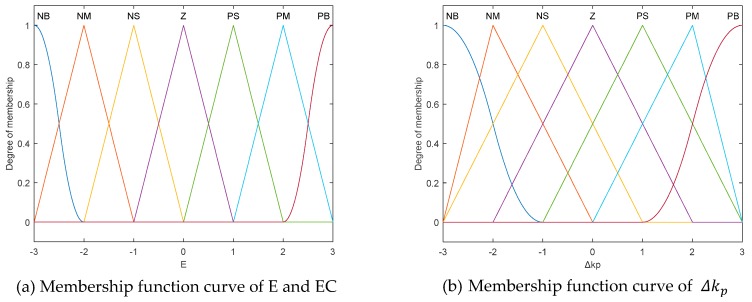

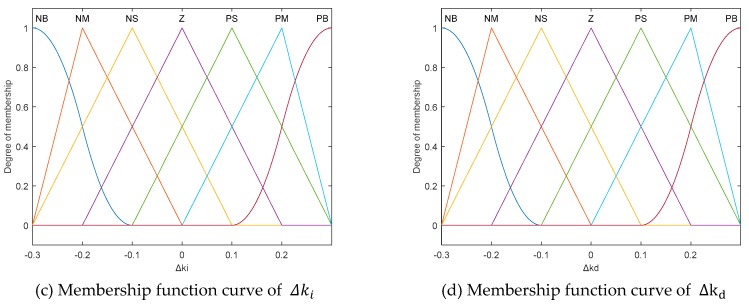
Membership function curve of input and output variables.

When the two are combined, the membership function curve of the input and output variables are shown in Figure 4. The PFID Controller’s fuzzy rules are shown in Table 1.

#### 3.2.2. Deblurring

According to the above protocol, the *e*, the *ec* and the resulting output variables are the fuzzy variables which need to be obtained by means of anti-ambiguity. Anti-modulus has a variety of ways, and commonly used is the area of the central method, maximum membership method and area integration method. Here we use the area center method to find the membership degree of the parameter increments $\Delta k_{p}$, $\Delta k_{i}$ and $\Delta k_{d}$ which are mathematically expressed as Equations (9)–(11).

(9) $$\mu \left(\Delta k_{p}\right)=min\{\mu_{NB}\left(e\right),\mu_{NB}\left(ec\right)\}$$

(10) $$\mu \left(\Delta k_{i}\right)=min\{\mu_{NB}\left(e\right),\mu_{NB}\left(ec\right)\}$$

(11) $$\mu \left(\Delta k_{d}\right)=min\{\mu_{NB}\left(e\right),\mu_{NB}\left(ec\right)\}$$

After the adjustment of the modulus and the rule, $\Delta k_{p}$, $\Delta k_{i}$ and $\Delta k_{d}$ can be obtained from the measurement of the queue length deviation *e* and the deviation rate *ec*. The mathematical formulas are as Equations (12)–(14).

(12) $$\Delta k_{p}=\frac{\sum_{j=1}^{49}\mu_{pj}\left(\Delta k_{p}\right)\cdot \Delta k_{pj}}{\sum_{j=1}^{49}\mu_{pj}\left(\Delta k_{p}\right)}$$

(13) $$\Delta k_{i}=\frac{\sum_{j=1}^{49}\mu_{ij}\left(\Delta k_{i}\right)\cdot \Delta k_{ij}}{\sum_{j=1}^{49}\mu_{ij}\left(\Delta k_{i}\right)}$$

(14) $$\Delta k_{d}=\frac{\sum_{j=1}^{49}\mu_{dj}\left(\Delta k_{d}\right)\cdot \Delta k_{dj}}{\sum_{j=1}^{49}\mu_{dj}\left(\Delta k_{d}\right)}$$

After calculating $\Delta k_{p}$, $\Delta k_{i}$ and $\Delta k_{d}$, we can get the parameters of the PID controller, that is, the final parameters of the FPID controller. By substituting these parameters into Equation (2), the updated packet loss rate *p* can be obtained.

### 3.3. Cuckoo Search for FPID Optimization

Compared with the traditional PID controller, the accuracy of the FPID controller is improved, but there is still room for further improvement. The packet loss rate calculated by FPID is not the optimal one, but tends to the optimal one [25]. To solve this problem, we use the efficient optimization ability of the Cuckoo search to optimize the packet loss rate which lead to get the final accurate value and control the queue length more accurately.

The Cuckoo search is an efficient meta heuristic optimization algorithm that mimics the way cuckoo birds hatch and reproduce [26]. In nature, cuckoos do not build their own nests. Instead, they seek out nests of other birds to lay their eggs and allow other birds to help them hatch. However, the host has a reasonable chance of finding the intruder’s eggs, at that point, the host may choose to either eliminate the eggs or abandon the nest. The Cuckoo search meets the following rules:

(1) A good host nest will be passed on to the next generation.
(2) The host has a fixed probability of discovery represented by $p_{a}$ such that $p_{a}\in \left[0,\;1\right]$.
(3) Each bird lays only one egg at a time and is placed in a randomly selected host nest with a fixed number of nests.

**Figure 5:**
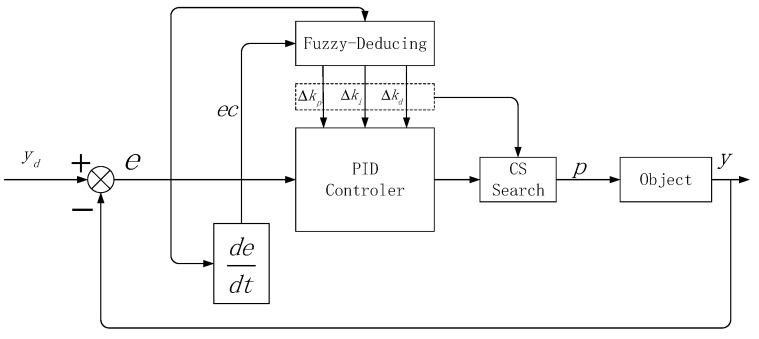
CFPID controller model.

Based on the above idea, in order to get the best packet loss rate, the message loss probability in the FPID model is taken as the target object and the model of the Cuckoo search-based mode and the CFPID controller (CFPID) is established, as shown in Figure 5.

The message drop probability p changes with the increment of parameter $\Delta k_{p}$, $\Delta k_{i}$ and $\Delta k_{d}$, and its expression is recorded as p(k). Taking p(k) as the objective function of cuckoo search and using Levis flight as the search path, the maximum value of message packet loss rate p(k) is calculated by updating and iterating.

The updated formula for the Cuckoo search path is as Equation (15).

(15) $$p_{k}^{\left(t+1\right)}=p_{k}^{\left(t\right)}+\alpha ⊗Levy\left(s,\lambda \right)$$

where, $p_{k}^{\left(t\right)}$ represents the position of the packet loss rate of the *K*th update at the *T*th iteration. Furthermore, α is the step factor which follows the normal distribution, so we set α=1. And Levy(s,λ) represents Levy-flight. A random walk through Levy- flight to get a random nest is formulated as Equation (16).

(16) $$Levy\left(s,\lambda \right)=\frac{\lambda \Gamma \left(\lambda \right)sin\left(\frac{\pi \lambda }{2}\right)}{\pi }\left(\frac{1}{s^{\lambda +1}}\right),\left(s\gg 0\right)$$

According to the above process, the Cuckoo search process is divided into four steps.

Step 1: initialize the population size *N*, the maximum number of iterations *M* and other parameters. In this manuscript, we set the population size *N* as 100, the maximum iteration number *M* as 100 and the probability of discovery as $p_{a}$ such that $p_{a}=\frac{\sum p_{k}^{\left(t\right)}}{t}$, and set its initial value as $p_{a}=1$. Taking packet loss rate p(k) as the objective function, update the formula for packet loss rate as Equation (17).

(17) $$p\left(k\right)=\left(k_{p0}+\Delta k_{p}\right)e\left(k\right)+\left(k_{i0}+\Delta k_{i}\right)T\overset{k}{\underset{j=0}{\sum }}e\left(i\right)+\left(k_{d0}+\Delta k_{d}\right)\left[\frac{e\left(k\right)-e\left(k-1\right)}{T}\right]$$

Step 2: Calculate the value of each position of p(k) and update the position using Levis’s flight Levy(s,λ). Then, calculate the objective function value R for this position and compare it with $p_{a}$. If $R>p_{a}$, then record *R* is the current optimal position and update the nest position, otherwise the position remains unchanged. 

Step 3: If the maximum number of iterations is satisfied, proceed to the next step or return to step 2.

Step 4: Output the global optimal solution and determine the best packet loss rate choice.

Pseudo code for the Cuckoo search is shown in Algorithm 1.
**Algorithm 1**. Cuckoo search algorithm**Input**: $\Delta k_{p}$, $\Delta k_{i}$, $\Delta k_{d}$, *M*, *N* **Output**: $p_{a}$
1: **while** (Number of iterations *m* < *M*) **do**
2:  Generate *N* random nest locations $k_{0,i}$ (i = 1,2,…,*N*)
3:  Calculate the objective function value of the initial nest $p\left(k_{0,i}\right)$
4:  (Levy-flights) Looking for a new nest site $k_{x,j}$ (i = 1,2,…,*N*)
5:  Calculate the value of the new nest objective function $p\left(k_{x,j}\right)$
6:  **if** ($p\left(k_{x,j}\right)$ > $p_{a}$) **then**
7:   $p_{a}\;$=$\;p\left(k_{x,j}\right)$
8:  **end if**
9:  With the best solution and the best location for the solution
10:  *m* ← *m* + 1
11: **end while**

The loss of packets can lead to an unacceptable increase in the number of packets, resulting in the loss of critical data when a certain amount is reached. In this method, the active packet loss starts earlier, the packet loss is planned and uniform, and the number of lost packets is kept at a low proportion, which can effectively avoid this problem.

The CFPID algorithm is an active queue management method with the ability of self-regulation. In this algorithm, the maximum number of iterations is *M*, the maximum population size is *N*, and there are no nested loops. So, the computational complexity of each iteration is less than O(M), and the spatial complexity of the Cuckoo search represented by O(N). In terms of active regulation, Levis Flight Levy(s,λ) is essentially a random walk with a fixed step, which is a Markoff chain. The next state position of the algorithm only depends on the current state and the discovery transition probability $p_{a}$. Most of the new solutions of the algorithm are derived from far-field randomization, and is far enough from the current optimal solution to ensure that the system does not fall into local optimality.

## 4. Simulation

In order to solve the problem of low accuracy in the computation of network congestion in WSNs, this manuscript presents a PID controller with a mold and a Cuckoo search. Furthermore, a mold and PID congestion control mechanism based on Cuckoo search is designed. The proposed CFPID algorithm is compared with the original PID algorithm and Immune clonal simulated annealing based Blue (IBLUE) algorithm [26] in terms of instantaneous queue length, packet loss rate and throughput which verified the feasibility of the improved algorithm.

In the comparison experiment, in order to make a more accurate and fair simulation comparison, the proportional, integral and differential parameters of the PID algorithm are set as the initial proportional, integral and differential parameters of the CFPID algorithm. So, set $k_{p0}=0.00129$, $k_{i0}=0.00222$ and $k_{d0}=0.00095$. IBLUE redefines the queue length and updates the packet loss rate, so the initial queue length and the expected queue length of CFPID algorithm are shared. Furthermore, the queue set value is 150 and the buffer is 220.

For performance comparison of CFPID, PID and IBLUE algorithms in WSN congestion control, this manuscript uses MATLAB R2018a. The experimental parameters are in Table 2.

### 4.1. Experimental Comparison of Instantaneous Queue Length

Firstly, the changes of message queue length for all the three algorithms are compared. Queue length can directly reflect the smooth transmission of message queue. If the queue length exceeds the expected value, congestion occurs and packet loss adjustments need to be made. However, the queue length is too small which will result in underutilization of network resource. Therefore, queue length should be close to the expected value. To this end, to show the effect of number of network nodes on queue length, we initially set the number of nodes to 100 for which, the length of the node queue changes as shown in Figure 5.

**Figure 6:**
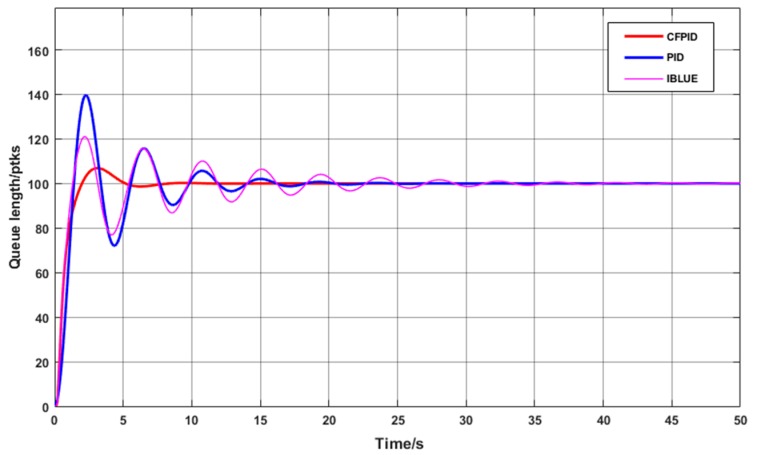
One-hundred instantaneous queue length curves for nodes.

When the data transfer starts, the message queue length of the node will increase rapidly and easily exceed the expected value of the queue length, which causes network congestion. Upon sensing the presence of network congestion, the control mechanism immediately begins to adjust the queue length. As can be seen from the graph in Figure 6, both PID and IBLUE algorithms start to show the control effect when the queue length reaches the highest point, and the convergence speed of the instantaneous queue length to the expected value is relatively slower in the later stage. In comparison, CFPID algorithm can control the length of the instantaneous queue to the expected value with the fastest convergence speed and the smallest oscillation amplitude, which shows the superiority of its control ability.

**Figure 7:**
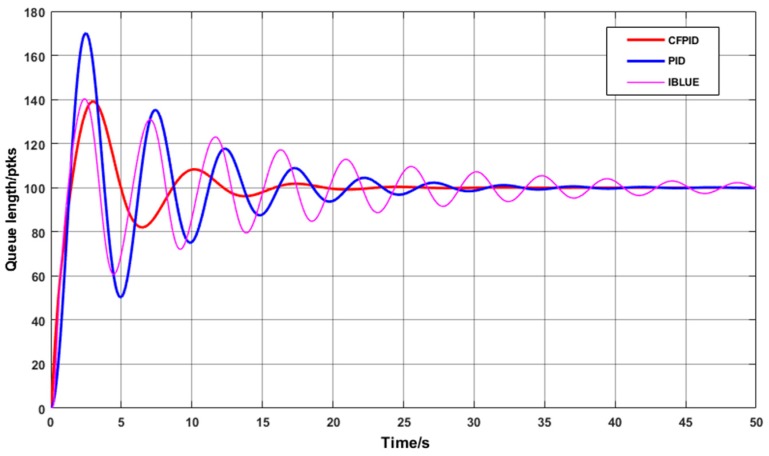
Two-hundred instantaneous queue length curves at node.

In order to observe the performance of CFPID algorithm under more complex and dynamic conditions, the number of nodes is adjusted from 100 to 200, which increases the data transmission load of the network and the workload of a single node. Then, we compared the message instantaneous queue length adjusted by CFPID, IBLUE and PID algorithms. The instantaneous queue length curve at 200 nodes is shown in Figure 7.

As the number of nodes increases, the volume of messages transferred and the workload of network nodes increase significantly. As can be seen in Figure 7, due to the increase of data volume, all the three control algorithms have obvious oscillation phenomenon, and the number of packets to be transmitted by a single node per unit time increases, the increase of oscillation amplitude and convergence time is emphasized, especially the change of oscillation amplitude of PID Algorithm. It can be seen in the Figure 7 that the oscillation amplitude of the instantaneous queue length controlled by CFPID algorithm is smaller than that of PID and IBLUE algorithms, and the convergence speed is the fastest.

### 4.2. Real-Time Packet Loss Rate Comparison

This manuscript compares the real-time packet loss rate of nodes for CFPID, PID and IBLUE algorithms. Generally, when the number of nodes increases, the number of packets to be transmitted also increases, resulting in the lengthening of the instantaneous queue length. When the length of the queue exceeds the expected value, the network congestion will be aggravated. Therefore, it is necessary to control the queue length by dropping a certain number of packets through the message dropping probability. The packet loss rate will change with the change in the instantaneous queue length, and the queue length will be controlled near the expected value. However, the unceasing change of packet loss rate will make the queue length change continuously, which will lead to the instability of the network. Therefore, the convergence rate and stability of the instantaneous packet loss rate will determine the performance of the network. 

**Figure 8:**
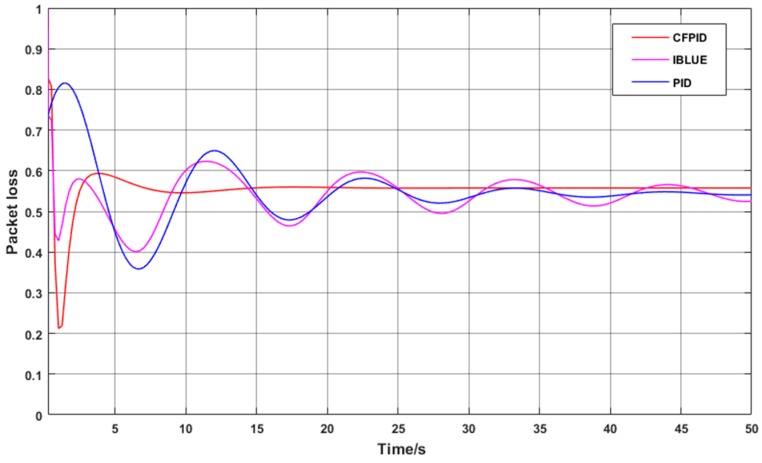
One-hundred node-time packet loss rate curves.

To this end, Figure 8 shows the change in the real-time packet loss rate when the initial number of nodes is set to 100. As can be seen from the graph, the CFPID algorithm can find out the danger more accurately when the message queue grows greatly. After a small amount of oscillation, CFPID will be the fastest to find the balance point, then only a small adjustment can be made to maintain the instantaneous queue length in a relatively stable state of health.

**Figure 9:**
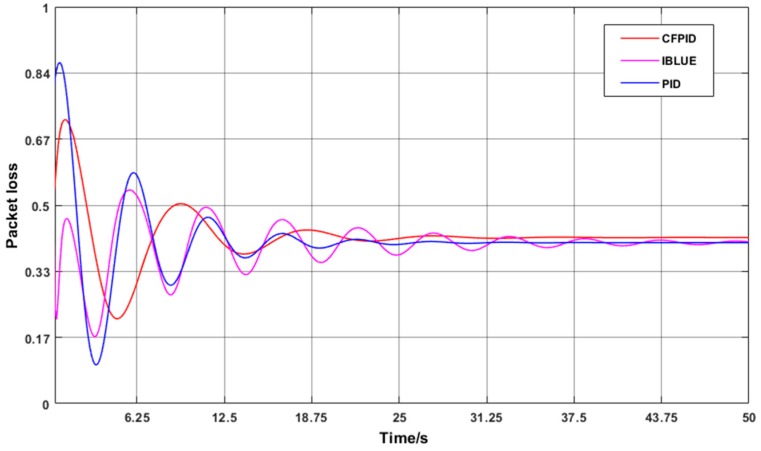
Two-hundred node-time packet loss rate curve.

In order to observe the convergence rate and stability of packet loss rate in complex scenarios, the number of nodes is increased to 200 which lead to further increase in the overall data volume and the number of packets to be transmitted by each node. We compare CFPID, IBLUE and PID congestion control mechanisms in complex case of packet loss rate changes. The change curve is shown in Figure 9.

**Figure 10:**
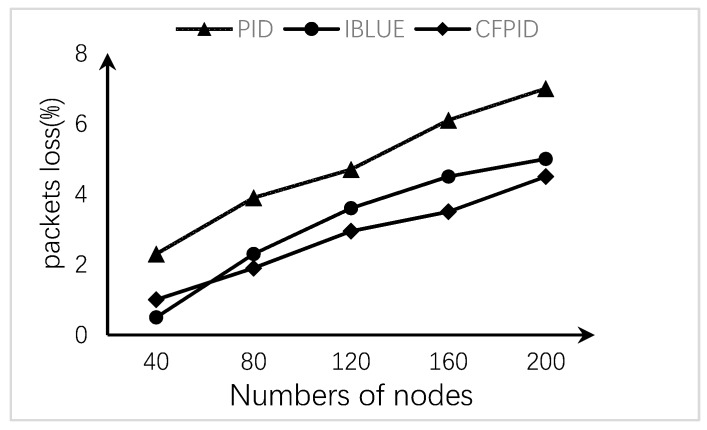
Packet loss rate curve.

The loss of data packets may cause the increase of the number of data packets and the loss of key data when the data reaches a certain amount. This may lead to the data information not being transmitted to the base station in time and cannot be accepted. In our method, the active packet loss starts earlier, the packet loss is planned and uniform, and the number of lost packets is kept at a low proportion, which can effectively avoid this problem. As the number of nodes increases, the packet loss rate curves for the three methods are shown in Figure 10.

As can be seen from the Figure 10, in the process of increasing the node distribution density, the increase of data makes the packet loss rate of the whole network present an upward trend. Among them, the loss rate of PID Algorithm has been kept at a high level, so that the key data cannot be transmitted in time, resulting in the decline of real-time data. The CFPID Algorithm can keep the gentle increasing slope, control the rate of packet loss in a relatively low proportion, and ensure the real-time of data.

### 4.3. Experimental Comparison of Throughput

**Figure 11:**
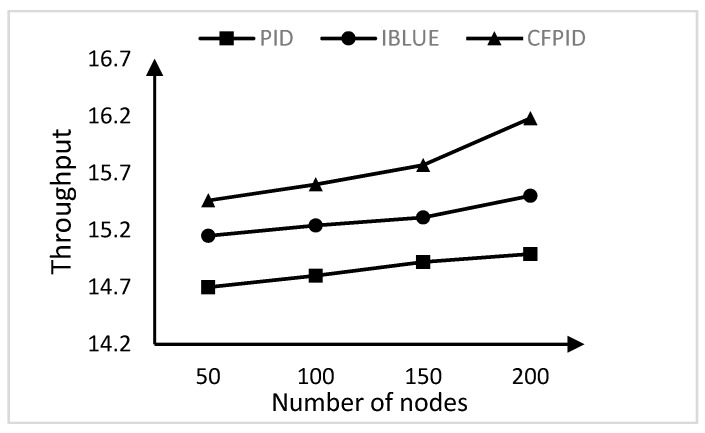
Throughput comparison.

Finally, we compare the average throughput of the three algorithms when the number of nodes increases. Throughput is an indicator of network performance. Increasing the number of nodes indirectly increases the amount of data passed. The throughput changes are shown in Figure 11.

Generally, an increase in the number of nodes will result in an increase in the messages transferred, decrease in the transmission time, and an increase in the throughput. It is clear from the Figure 11 that the throughput of the three congestion control algorithms increases with the increase of the number of nodes. When the number of nodes is small, CFPID algorithm has the highest throughput. With the increase of the number of nodes, the throughput of IBLUE and PID will be surpassed by CFPID, which shows the superiority of CFPID in terms of stability, and adaptive adjustment in complex cases. As can be seen from the Figure 11, the throughput of the method presented in this manuscript is improved by 4%–8% compared with the comparison method.

### 4.4. Comparison of Running Time under Different Number of Nodes

**Figure 12:**
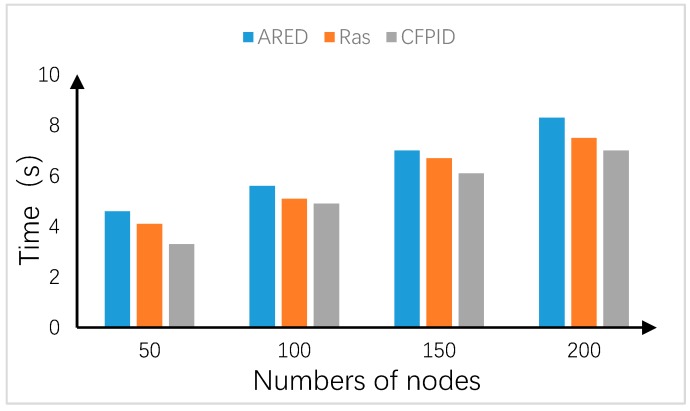
Running time comparison.

In this manuscript, our designed CFPID scheme is compared with ARED [27] and Ras algorithms [28] in running time. Generally, a gradually increase in the density of network nodes increases the number of nodes and indirectly increase the overall network data. The running time of the algorithm can directly reflect the load capacity of the algorithm in complex environment. In addition, the short running time can reduce the loss of network resources. The run-time changes are shown in Figure 12. 

As can be seen from the Figure 12, as the number of sensor nodes in the network increases, the data received by the nodes increases greatly, and the running time of the algorithm also increases. Among them, the running time of CFPID algorithm is the least. This is because the convergence speed of the algorithm is fast and it can find the appropriate packet loss scheme in the shortest time. In addition, CFPID algorithm can control the degree of congestion, so it can effectively save the energy of sensor nodes and prolong the lifetime of sensor network effectively. At the same time, an increase in the number of nodes increases the complexity of the environment but CFPID algorithm can still show good adaptability which reflects the stability of this algorithm.

## 5. Conclusions

In this manuscript, a new congestion control mechanism for WSNs is proposed, which combines fuzzy and PID control with Cuckoo search control. The real-time packet loss rate is calculated by PID controller to control the instantaneous queue length of nodes. The parameters of PID are adjusted by mold and control to improve the self-adaptive optimization ability, and the corresponding mold and rules are given. Finally, the Cuckoo search is used to optimize the accuracy of packet loss rate globally. In future, more meta-heuristic optimization algorithms can be combined to optimize the optimization rate of the Cuckoo search and the mode, and the PID controller.

## Figures and Tables

**Table 1:** Table of FPID mode and rule.

$\Delta k_{p}/$ $\Delta k_{i}/$ $\Delta k_{d}$		ec						
		NB	NM	NS	Z	PS	PM	PB
**e**	**NB**	PB/NB/PS	PB/NB/NS	PM/NM/NB	PM/NM/NB	PS/NS/NB	Z/Z/NM	Z/Z/PS
	**NM**	PB/NB/PS	PB/NB/NS	PM/NM/NB	PS/NS/NM	PS/NS/NM	Z/Z/NS	NS/Z/Z
	**NS**	PM/NB/Z	PM/NM/NS	PM/NS/NM	PS/NS/NM	Z/Z/NS	NS/PS/NS	NS/PS/Z
	**Z**	PM/NM/Z	PM/NM/NS	PS/NS/NS	Z/Z/NS	NS/PS/NS	NM/PM/NS	NM/PM/Z
	**PS**	PS/NM/Z	PS/NS/Z	Z/Z/Z	NS/PS/Z	NS/PS/Z	NM/PM/Z	NM/PB/Z
	**PM**	PS/Z/PB	Z/Z/PS	NS/PS/PS	NM/PS/PS	NM/PM/Z	NM/PB/PS	NB/PB/PB
	**PB**	Z/Z/PB	Z/Z/PM	NM/PS/PM	NM/PM/PM	NM/PM/PS	NB/PB/PS	NB/PB/PB

**Table 2:** Table of simulation parameters.

Simulation Parameter	Prescribed Value
Simulated area	100*100
Number of nodes	100/200
Simulation time	50
Expected Queue length	100
